# Factors Related to Prostate-Specific Antigen–Based Prostate Cancer Screening in Primary Care: Retrospective Cohort Study of 120,587 French Men Over the Age of 50 Years

**DOI:** 10.2196/10352

**Published:** 2018-10-23

**Authors:** Cédric Rat, Heloise Schmeltz, Sylvain Rocher, France Nanin, Aurélie Gaultier, Jean-Michel Nguyen

**Affiliations:** 1 Department of General Practice Faculty of Medicine University of Nantes Nantes France; 2 Team 2 Unit 1232 French National Institute of Health and Medical Research Nantes France; 3 French Health Insurance System Nantes France; 4 Department of Epidemiology and Biostatistics Nantes University Hospital Nantes France

**Keywords:** prostate cancer, screening, prostate-specific antigen testing, general practice, primary care

## Abstract

**Background:**

International guidelines recommend avoiding prostate-specific antigen (PSA)-based prostate cancer screening in the elderly when life expectancy is less than 10 years. For younger men, most recommendations encourage a shared decision-making process taking into account patient comorbidities.

**Objective:**

The objective was to assess the performance of PSA-based prostate cancer screening in men older than 74 years and assess whether the presence (vs absence) of comorbidities was related to the performance of PSA testing in younger men aged 50 to 74 years who were eligible for screening.

**Methods:**

We analyzed data from the French national health care database (Loire-Atlantique geographic area). We reported the follow-up of two cohorts of men from April 1, 2014, to March 31, 2016: 22,480 men aged over 74 years and 98,107 men aged 50 to 74 years. We analyzed whether these patients underwent PSA testing after 2 years of follow-up and whether PSA testing performance was related to the following patient-related variables: age, low income, proxy measures indicative of major comorbidities (repeated ambulance transportation, having one of 30 chronic diseases, taking 5 or more drugs per day), or proxy measures indicative of specific comorbidities (cancer diseases, cardiovascular diseases, or psychiatric disorders). Statistical analysis was based on a multivariate mixed-effects logistic regression.

**Results:**

The proportion of patients who underwent a PSA-based screening test was 41.35% (9296/22,480) among men older than 74 years versus 41.05% (40,275/98,107) among men aged 50 to 74 years. The following factors were associated with less frequent PSA testing in men older than 74 years—age (odds ratio [OR] 0.89, 95% CI 0.88-0.89), low income (OR 0.18, 95% CI 0.05-0.69), suffering from a chronic disease (OR 0.82, 95% CI 0.76-0.88), repeated ambulance transportation (OR 0.37, 95% CI 0.31-0.44), diabetes requiring insulin (OR 0.51, 95% CI 0.43-0.60), dementia (OR 0.68, 95% CI 0.55-0.84), and antipsychotic treatment (OR 0.62, 95% CI 0.51-0.75)—whereas cardiovascular drug treatment was associated with more frequent PSA testing (OR 1.6, 95% CI 1.53-1.84). The following factors were associated with less frequent PSA testing in men aged 50 to 74 years—low income (OR 0.61, 95% CI 0.55-0.68); nonspecific conditions related to frailty: suffering from a chronic disease (OR 0.80, 95% CI 0.76-0.83), repeated ambulance transportation (OR 0.29, 95% CI 0.23-0.38), or chronic treatment with 5 or more drugs (OR 0.89, 95% CI 0.83-0.96); and various specific comorbidities: anticancer drug treatment (OR 0.67, 95% CI 0.55-0.83), diabetes requiring insulin (OR 0.55, 95% CI 0.49-0.61), and antiaggregant treatment (OR 0.91, 95% CI 0.86-0.96)—whereas older age (OR 1.07, 95% CI 1.07-1.08) and treatment with other cardiovascular drugs (OR 2.23, 95% CI 2.15-2.32) were associated with more frequent PSA testing.

**Conclusions:**

In this study, 41.35% (9296/22,480) of French men older than 74 years had a PSA-based screening test. Although it depends on patient comorbidities, PSA testing remains inappropriate in certain populations.

## Introduction

Prostate-specific antigen (PSA)-based screening for prostate cancer is challenging for both clinicians and policy makers [1-2]. Based on the most recent evidence [3-6], the US Preventive Services Task Force modified its recommendation in 2017 [7-8]. While the previous 2012 version recommended against screening regardless of patient age, the latest draft is consistent with previous French and Canadian guidelines published in 2014 and 2015 [8-11].

These guidelines recommend avoiding screening in the elderly. The evidence shows that prostate cancer is slow growing, the 10-year survival rate is higher than 95%, and rates of overdiagnosis are elevated in older men [12]. In total, there is a consensus that screening may result in more harm than benefit in the elderly [8-11,13-14], and French guidelines recommend avoiding screening in men older than 74 years because they have a life expectancy shorter than 10 years [9-10].

Most recommendations encourage an individual approach for men aged 50 to 69 years [8-11,13-14] based on a shared decision-making process [15-16]. Thresholds provided by the US Preventive Services Task Force define a narrower group, limiting eligibility for screening to men aged 55 to 69 years, while French guidelines consider men aged 50 to 74 years [8-10]. However, the philosophy of these recommendations is similar, reporting that clinicians should inform eligible men about the potential benefits and harms of PSA-based screening. Screening probably offers a small benefit of reducing the probability of dying of prostate cancer, but many men will experience harms from screening, including false-positive results that require additional testing, possible prostate biopsy, overdiagnosis, overtreatment, and possible treatment complications such as incontinence and impotence [7-8,17-18].

In France, as in various other countries, general practitioners (GPs) prescribe the majority of PSA tests [19]. Various tools and decision aids have been developed to help GPs share and personalize the screening decision with their patients, integrating eligible men’s values and medical characteristics [20-22]. From a medical perspective, based on scientific evidence, patients with an expected survival of less than 10 years should remain unscreened. To our knowledge, there is no algorithm allowing a robust assessment of survival for an individual, but screening decisions should at a minimum be related to patients’ comorbidities [8-14,18]. Implementation of a shared decision-making process is difficult, and previous authors reported that 41% of French men underwent prostate cancer screening based on PSA between 2008 and 2010 [23-24]. Screening decisions might mainly depend on GPs’ primary goals [2]. It is unclear whether a shared decision-making process would lead to decisions based on scientific evidence or whether the patient might make a decision without any consideration of medical factors, such as comorbidities or life expectancy.

The first objective of this study was to assess the inappropriate performance of PSA testing in men older than 74 years. The secondary objective was to assess whether the presence (vs absence) of comorbidities was related to the performance of PSA testing in younger men aged 50 to 74 years who were eligible for screening.

## Methods

### Design, Setting, and Patients

We used the French national health care system’s administrative database to collect longitudinal follow-up data from two cohorts of male patients. Access to the anonymized data was provided by the national health care insurance services, which participated in the study after receiving permission from the health care insurance authorities.

All patients eligible for the study lived on the west coast of France in the Loire-Atlantique geographical area (1,346,592 inhabitants), were over the age of 50 years, and were affiliated with one of the 1183 GPs who practiced in the geographical area at the beginning of the study (April 1, 2014). Patients who changed their GP during the study period were excluded from the analysis regardless of the reason (ie, retirement, death, or career move). Patients were excluded if (1) they were currently being treated for prostate cancer using any of the following drugs: abiraterone, bicalutamide, cyproterone, degarelix, diethylstilbestrol, enzalutamide, flutamide, goserelin, leuprorelin, nilutamide, or triptorelin; (2) PSA testing was prescribed by a urologist (to avoid the inclusion of patients with prostate cancer); or (3) the patient died during the study period.

Patients were grouped into 2 cohorts: (1) 50- to 74-year-old patients eligible for prostate cancer screening and (2) patients older than 74 years for whom screening should be avoided.

### Main Outcome Measure

We analyzed whether the patients had undergone PSA testing during the 2-year follow-up period using the French Classification of Medical Acts (code 7318), and the rate of patients screened during the study period was calculated for the 2 cohorts.

### Data Extraction From National Health Care Insurance Records

Patient characteristics were collected as follows: age, whether the patient had a low income (defined as an annual income less than 8593 € [US $9992] for an individual or less than 12889 € [US $14,925] for a couple), and proxy measures indicative of major comorbidities. Frail individuals were first identified using the following nonspecific proxy measures: whether the patient required repeated ambulance transportation during the study period (6 times or more), whether he had one of 30 chronic diseases leading to reimbursements for facilities, and whether his chronic treatment included 5 or more drugs per day. Frail individuals were also identified by the following specific comorbidities (the related proxy measures are provided in parentheses):

Cancer diseases (31 anticancer drugs and tumor-related factors such as carcinoembryonic antigen, CA-19-9 antigen, and squamous cell carcinoma–related antigen)

- Cardiovascular diseases (number of cardiovascular drugs used for chronic treatment and chronic insulin use)
- Psychiatric disorders such as dementia (anticholinesterasic treatment or memantine) or major psychiatric disorders (chronic treatment with either antipsychotics or more than 3 psychiatric drugs)
- Variables indicative of other comorbidities (oxygen at home, more than 8 serum urea and creatinine tests during the 2-year study period, or more than 4 alpha-fetoprotein tests during the study period)

Patients with clinical symptoms of benign prostate hyperplasia (dysuria or prostatism) were identified using a proxy measure—treatment with one of the following drugs: alfuzosin, doxazosin, dutasteride, finasteride, prazosin, Pygeum africanum, Serenoa repens, silodosin, tamsulosin, and terazosin.

### Statistical Analysis

We first reported the patient and GP characteristics. All analyses were then performed using R version 3.3.1 statistical software (R Foundation for Statistical Computing) and SAS version 9.4 (SAS Institute Inc). For all statistical analyses, the patient was considered the statistical unit. Descriptive statistics were reported using means, standard deviations, and frequency distributions. A first analysis focused on patients older than 74 years, for whom screening should be avoided. A second analysis was performed for patients aged 50 to 74 years, for whom screening should be based on a shared decision-making process. Bivariate analysis was used to compare men who had a PSA test to men who did not using a chi-square test or Student *t* test. Variables with a *P*<.20 were entered into the logistic regression model. A backward procedure based on Akaike information criterion minimization was then performed on these data in order to select the discriminant patients’ characteristics. Finally, we adjusted the previous selected model on the general practitioner factor as a random effect in a mixed model. An alpha level of .05 was chosen to assess statistical significance.

### Ethics Statement

Ethics approval and specific written informed consent from the participants were not required for this retrospective cohort study performed in France.

## Results

### Retrospective Cohort Constitution

In total, 129,392 men aged over 50 years were affiliated with GPs practicing in the Loire-Atlantique geographical area at the beginning of the study. However, 8805 of these patients were excluded for the following reasons: 774 individuals died during the study period, 6829 patients’ GP stopped practicing during the study period, and 1202 men underwent prostate cancer–related treatment. In total, the study reported the 2-year follow-up of 120,587 men who were affiliated with 968 GPs: 98,107 were aged 50 to 74 years and 22,480 were older than 74 years.

### Patient and General Practitioner Characteristics

**Figure 1:**
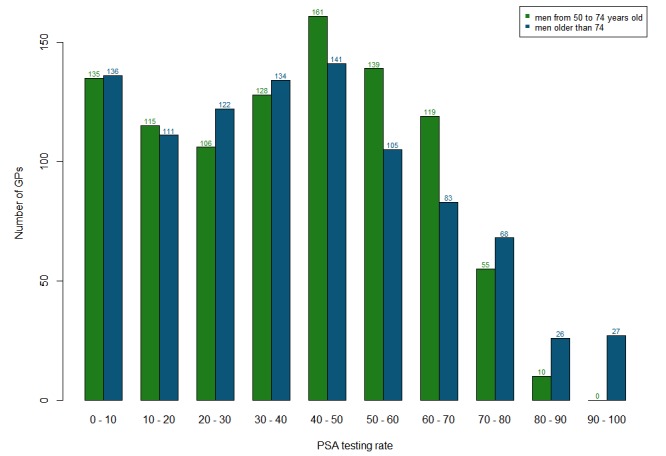
Distribution of prostate-specific antigen (PSA) testing performance rates according to general practitioner (GP; defined as the proportion of patients who underwent PSA testing in each GP’s patient panel).

The mean age of the GPs was 53.1 (SD 9.3) years, and 591 (61.1%) were men. Among the GPs, 56.2% (544/968) had an urban practice, 36.5% (353/968) had a semirural practice, and 7.3% (71/968) had a rural practice in cities with fewer than 2000 inhabitants. The mean number of male patients older than 50 years who visited the physicians during the study period was 124.6 (SD 72.3). Figure 1 shows that the probability of undergoing a PSA screening test, both in the cohort of men aged 50 to 74 years and in the cohort of men older than 74 years, varied depending on which physician a patient consulted*.*

The mean patient age was 64.6 (SD 10.5) years. A low income was identified in 1.96% (2367/120,587) of all patients. A total of 36.21% (43,663/120,587) of all patients suffered from one of 30 severe chronic diseases related to reimbursement of facilities. Other characteristics provided insights into frailty and comorbidities (Table 1).

### Proportion of Patients Who Underwent Prostate-Specific Antigen Testing During the 2-Year Study Period

The proportion of patients who received a PSA test during the 2-year study period was not lower in the cohort of men older than 74 years than in the cohort of men aged 50 to 74 years: 41.35% (9296/22,480, 95% CI 40.7-42.0) vs 41.05% (40,275/98,107, 95% CI 40.7-41.4).

### Factors Associated With Prostate-Specific Antigen Testing in the Cohort of Men Aged Older Than 74 Years

In the cohort of men older than 74 years, the following factors were associated with PSA testing: (1) age (odds ratio [OR] 0.89, 95% CI 0.88-0.89; (2) low income (OR 0.18, 95% CI 0.05-0.60); (3) nonspecific conditions related to frailty: chronic disease (OR 0.82, 95% CI 0.76-0.88) and repeated ambulance transportation (OR 0.37, 95% CI 0.31-0.44); and (4) various specific comorbidities: diabetes requiring insulin (OR 0.51, 95% CI 0.43-0.60), dementia (OR 0.68, 95% CI 0.55-0.84), and antipsychotic treatment (OR 0.62, 95% CI 0.51-0.75; Table 2). Higher screening rates were observed in patients treated for cardiovascular diseases (compared to no cardiovascular treatment), and these rates remained high regardless of the number of drugs taken: 1 or 2 cardiovascular drugs (OR 1.6, 95% CI 1.53-1.84), 3 or 4 cardiovascular drugs (OR 1.73, 95% CI 1.57-1.91), or 5 or more cardiovascular drugs (OR 1.64, 95% CI 1.46-1.84). The following patient characteristics were not significantly correlated with lower PSA testing: having oxygen at home, more than 8 urea/creatinine tests during the study period, and more than 4 alpha-fetoprotein tests during the study period.

**Table 1:** Patient characteristics in 2 age-based cohorts of patients: 50 to 74 years and older than 74 years.

Characteristics			Total patients (N=120,587)	Patients aged 50 to 74 years (n=98,107)	Patients older than 74 years (n=22,480)
Age in years, mean (SD)			64.6 (10.5)	60.7 (6.9)	81.6 (5.1)
Low socioeconomic status^a^, n (%)			2367 (1.96)	2344 (2.39)	23 (0.10)
**Frail individual, n (%)**					
	Chronic disease status		43,663 (36.21)	29,875 (30.45)	13,788(61.33)
	Repeated ambulance transportation		929 (0.77)	391 (0.40)	538 (2.39)
	Chronic treatment with ≥5 drugs		19,212 (15.93)	12,277 (12.51)	6935 (30.85)
Cancer disease (treated with anticancer drug), n (%)			780 (0.65)	515 (0.52)	265 (1.18)
**Cardiovascular disease, n (%)**					
	**Number of cardiovascular drugs**				
		0	54,844 (45.48)	50,678 (51.66)	4166 (18.53)
		1-2	32,497 (26.95)	25,180 (25.67)	7317 (32.55)
		3-4	20,235 (16.78)	13,540 (13.80)	6695 (29.78)
		5 or more	1301 (10.79)	8709 (8.88)	4302 (19.14)
	Treated with insulin		3116 (2.58)	2194 (2.24)	922 (4.10)
	Treated with antiaggregant		27,836 (23.08)	18,089 (18.44)	9747 (43.36)
Dementia (treated with anticholinesterase therapy), n (%)			699 (0.58)	126 (0.13)	573 (2.55)
Psychiatric disorder (treated with antipsychotic therapy), n (%)			3237 (2.68)	2541 (2.59)	696 (3.10)
Urology (treatment for benign prostate hyperplasia), n (%)			14,849 (12.31)	8863 (9.03)	5986 (26.63)
**Other variables indicative of comorbidities, n (%)**					
	Oxygen at home		4932 (4.09)	3999 (4.08)	933 (4.15)
	>8 urea/creatinine tests during the study period		1281 (1.06)	1042 (1.06)	239 (1.06)
	>4 alpha-fetoprotein tests during the study period		255 (0.21)	199 (0.20)	56 (0.25)

^a^Defined as an annual income less than 8593 € (US $9992) for an individual or less than 12889 € (US $14,925) for a couple.

**Table 2:** Factors related to the performance of prostate-specific antigen testing in a cohort of French men older than 74 years (mixed-effects multivariate logistic regression with general practitioner as a random effect).

Characteristics			Proportion of patients screened using PSA^a,b^ (%)	Crude odds ratio (95% CI)^c^	*P* value^d^	Adjusted odds ratio (95% CI)^e^	*P* value^f^
Age in years			N/A^g^	0.89 (0.89-0.90)	<.001	0.89 (0.88-0.89)	<.001
Low socioeconomic status^h^			13.04	0.25 (0.07-0.89)	.03	0.18 (0.05-0.69)	.01
**Frail individual**							
	Chronic disease status		39.61	0.80 (0.75-0.85)	<.001	0.82 (0.76-0.88)	<.001
	Repeated ambulance transportation		18.77	0.28 (0.22-0.36)	<.001	0.42 (0.33-0.54)	<.001
	Chronic treatment with ≥5 drugs		41.50	0.98 (0.92-1.05)	.57	N/A	N/A
Cancer disease (treated with anticancer drug)			39.62	0.88 (0.67-1.16)	.36	N/A	N/A
**Cardiovascular disease**							
	**Number of cardiovascular drugs**						
		0	35.93	Reference	N/A	Reference	N/A
		1-2	43.53	1.44 (1.32-1.57)	<.001	1.68 (1.53-1.84)	<.001
		3-4	42.63	1.37 (1.25-1.49)	<.001	1.73 (1.57-1.91)	<.001
		5 or more	40.91	1.24 (1.13-1.37)	<.001	1.64 (1.46-1.84)	<.001
	Treated with insulin		27.22	0.49 (0.42-0.58)	<.001	0.62 (0.51-0.75)	<.001
	Treated with antiaggregant		41.43	0.99 (0.93-1.05)	.80	N/A	N/A
Dementia (treated with anticholinesterase therapy)			25.83	0.44 (0.36-0.54)	<.001	0.68 (0.55-0.84)	<.001
Psychiatric disorder (treated with antipsychotic therapy)			29.02	0.49 (0.40-0.58)	<.001	0.62 (0.51-0.75)	<.001
**Other variables indicative of comorbidities**						N/A	N/A
	Oxygen at home		43.30	1.12 (0.96-1.29)	.14		
	>8 urea/creatinine tests during the study period		43.93	1.25 (0.94-1.66)	.12		
	>4 alpha-fetoprotein tests during the study period		41.07	1.06 (0.58-1.91)	.85		

^a^Prostate-specific antigen.

^b^n=22,480.

^c^General practitioner as a random effect; bivariate analysis.

^d^*P* value for crude odds ratio.

^e^General practitioner as a random effect; multivariate analysis; adjusted on the variable “treatment for benign prostate hyperplasia.”

^f^*P* value for adjusted odds ratio.

^g^N/A: not applicable.

^h^Defined as an annual income less than 8593 € (US $9992) for an individual or less than 12889 € (US $14,925) for a couple.

### Factors Associated With Prostate-Specific Antigen Testing in the Cohort of Men Aged 50 to 74 Years

In the cohort of men aged 50 to 74 years, the following factors were associated with less frequent PSA testing: (1) low income (OR 0.61, 95% CI 0.55-0.68); (2) nonspecific conditions related to frailty: chronic disease (OR 0.80, 95% CI 0.76-0.83), repeated ambulance transportation (OR 0.29, 95% CI 0.23-0.38), or chronic treatment with more than 5 drugs (OR 0.89, 95% CI 0.83-0.96); and (3) various specific comorbidities: anticancer drug treatment (OR 0.67, 95% CI 0.55-0.83), diabetes requiring insulin (OR 0.55, 95% CI 0.49-0.61), and antiaggregant treatment (OR 0.91, 95% CI 0.86-0.96; Table 3). Higher screening rates were observed in patients treated for cardiovascular diseases (compared to no cardiovascular treatment), and these rates remained high regardless of the number of drugs taken: 1 or 2 cardiovascular drugs (OR 2.23, 95% CI 2.15-2.32), 3 or 4 cardiovascular drugs (OR 2.61, 95% CI 2.46-2.77), or 5 or more cardiovascular drugs (OR 2.64, 95% CI 2.40-2.91). Older age was also associated with more frequent PSA testing (OR 1.07, 95% CI 1.07-1.08). The following patient characteristics were not significantly correlated with lower PSA testing: having oxygen at home, having more than 8 urea/creatinine tests during the study period, having more than 4 alpha-fetoprotein tests during the study period, and treatment with psychotropic drugs.

**Table 3:** Factors related to the performance of prostate-specific antigen testing in a cohort of French men aged 50 to 74 years (mixed-effects multivariate logistic regression with general practitioner as a random effect).

Characteristics			Proportion of patients screened using PSA^a,b^ (%)	Crude odds ratio (95% CI)^c^	*P* value^d^	Adjusted odds ratio (95% CI)^e^	*P* value^f^
Age in years			N/A^g^	1.09 (1.09-1.10)	<.001	1.07 (1.07-1.07)	<.001
Low socioeconomic status^h^			23.55	0.43 (0.39-0.48)	<.001	0.61 (0.55-0.68)	<.001
**Frail individual**							
	Chronic disease status		46.28	1.42 (1.37-1.46)	<.001	0.79 (0.76-0.83)	<.001
	Repeated ambulance transportation		24.55	0.42 (0.33-0.53)	<.001	0.29 (0.23-0.38)	<.001
	Chronic treatment with ≥5 drugs		50.46	1.66 (1.59-1.73)	<.001	0.89 (0.83-0.96)	.002
Cancer disease (treated with anticancer drug)			37.48	0.83 (0.70-1.01)	.06	0.67 (0.55-0.83)	<.001
**Cardiovascular disease**							
	**Number of cardiovascular drugs**						
		0	31.11	Reference	N/A	Reference	N/A
		1-2	51.31	2.66 (2.56-2.75)	<.001	2.23 (2.15-2.32)	<.001
		3-4	53.69	2.98 (2.86-3.12)	<.001	2.61 (2.46-2.77)	<.001
		5 or more	49.59	2.46 (2.34-2.59)	<.001	2.64 (2.40-2.91)	<.001
	Treated with insulin		38.51	0.87 (0.79-0.96)	.005	0.55 (0.49-0.61)	<.001
	Treated with antiaggregant		51.17	1.80 (1.73-1.86)	<.001	0.91 (0.86-0.96)	.001
Dementia (treated with anticholinesterase therapy)			53.17	1.46 (0.99-2.17)	.06	N/A	N/A
Psychiatric disorder (treated with antipsychotic therapy)			35.73	0.79 (0.72-0.86)	<.001	N/A	N/A
**Other variables indicative of comorbidities**						N/A	N/A
	Oxygen at home		41.79	1.02 (0.95-1.10)	.51		
	>8 urea/creatinine tests during the study period		40.50	0.98 (0.85-1.12)	.72		
	>4 alpha-fetoprotein tests during the study period		41.71	1.02 (0.75-1.38)	.92		

^a^Prostate-specific antigen.

^b^ n=98,107.

^c^General practitioner as a random effect; bivariate analysis.

^d^*P* value for crude odds ratio.

^e^General practitioner as a random effect; multivariate analysis; adjusted on the variable “treatment for benign prostate hyperplasia.”

^f^*P* value for adjusted odds ratio.

^g^N/A: not applicable.

^h^Defined as an annual income less than 8593 € (US $9992) for an individual or less than 12889 € (US $14,925) for a couple.

## Discussion

### Principal Findings

In our study, the proportion of patients who underwent PSA testing during the 2-year study period was not lower in the cohort of men older than 74 years than in the cohort of men aged 50 to 74 years: 41.35% (95% CI 40.7-42.0) vs 41.05% (95% CI 40.7-41.4). The following factors associated with less frequent PSA testing were similar in men older than 74 years and in men aged 50 to 74 years—chronic disease, repeated ambulance transportation, diabetes, psychiatric disorders, and low income—whereas being treated with cardiovascular drugs was associated with more frequent PSA testing. Although PSA testing depends on patients’ comorbidities, test performance remains inappropriate in certain populations: elderly patients should not be screened, particularly when they have dementia or major comorbidities. The reasons why lower screening rates are observed among patients with insulin or among patients with a low income are unclear.

The proportion of patients who underwent PSA screening in our study conducted in France is comparable to previous evaluations provided by other French authors [23-24] but is much higher than the proportions reported by authors from other countries [25-32]. Among men aged 50 to 74 years, the observed 41.05% rate of French patients who had undergone PSA screening is comparable to the rates of participation in systematic screenings for colorectal cancer or breast cancer. In France, participation in colorectal cancer screening is lower than 30% [33], and participation in breast cancer screening is 51.5% [34]. Although prostate cancer screening is not recommended in the elderly, the PSA blood test was performed as frequently in patients older than 74 years as in younger men. A possible reason is that this test is highly acceptable to patients [35]. Other reasons may include positive attitudes toward screening, such as considering it a favorable option, or physicians’ fear of legal consequences related to diagnostic delay [28,36]. Another reason might be that prescribing PSA screening might be easier than explaining the reason why this test should not be performed. As French GPs practice in a pay-per-act system, prescribing PSA testing might decrease the time spent on a consultation compared with a shared decision-making process leading to abstention. Finally, various other factors probably limit shared decision-making implementation in primary care practices in France: deficiencies in initial medical education and law medical demography as well as the lack of an interactive decision-making aid to support GPs and patients when making prostate cancer screening decisions.

PSA testing occurred less frequently in frail patients and patients with major comorbidities. This finding is consistent with international guidelines and recommendations suggesting that life expectancy should be considered before recommending screening [8,17]. Surprisingly, more than 30% of patients treated with anticholinesterase therapies and 20% of patients with 6 or more ambulance transportations during the study period underwent PSA-based prostate screening. While all guidelines recommend avoiding screening in patients with a life expectancy of less than 10 years [8,17,36-37], previous authors have also reported inappropriate screening practices in vulnerable patients [38-40]. These results emphasize that integrating life expectancy into medical decisions remains a challenge for primary care physicians [41-42].

Three populations were screened less frequently, although they had no clear link with a shorter life expectancy: patients treated with insulin, patients treated with antipsychotic medications, and patients with low incomes. Various authors have reported low participation in preventive procedures in patients treated with antipsychotic medications and patients with low incomes [43-44]. Lower PSA testing in deprived patients has been reported in other countries [30-45] and might be related to lower access to health care in these populations. We assume, however, that this result might paradoxically be appropriate for prostate cancer screening; physicians might concentrate their time and energy on other health problems in patients suffering from various diseases.

Patients treated for cardiovascular diseases underwent PSA screening more frequently than other patients. It is notable that this result is consistent with previous international findings, although the reasons remain unclear. One reason for this finding might be that these patients consult their physicians more frequently [46], at least for prescription refills, and may have more frequent blood analyses [30,47]. Another possible reason is that these patients might have experienced the positive impact of medical interventions, which might favor positive attitudes toward screening proposals.

### Strengths and Limitations

This database study had many strengths. First, the study design allowed for the inclusion of a large number of patients and GPs; thus, our results should be representative of PSA performance in the general population and have high generalizability. Second, the data were extracted from the national health care insurance system database. We did not collect reported data from surveys, avoiding any related bias (eg, response bias or social desirability bias). Finally, the inclusion of a large number of patients permitted the analysis of specific conditions corresponding to a low proportion of patients.

This study also had limitations. First, the database did not contain clinical information allowing a determination of whether the PSA blood analysis had been prescribed as a result of clinical symptoms or as part of a screening practice. Second, we focused on PSA tests prescribed by GPs. Although they are a minority in the French health care system, asymptomatic patients might also consult urologists and be prescribed a PSA test for prostate cancer screening. Third, another weakness of the study was the use of proxy measures (comorbidities deduced from the types of drugs administered during the study period); although the use of proxy measures is common, the proxy measures used to assess frailty in this study had not been validated in previous studies.

### Conclusion

This study provided insight into the wide variations in prostate cancer screening using PSA. This study demonstrated that PSA testing is much more frequent in France than in other countries. Although there is a consensus that screening should be avoided in patients with a life expectancy less than 10 years, PSA testing remained very frequent in patients older than 74 years. This study also demonstrated that physicians considered patient conditions but PSA testing remained inappropriate in certain populations such as patients with dementia.

## Abbreviations

GP: general practitioner
OR: odds ratio
PSA: prostate-specific antigen
